# Drinking Moderately and Pregnancy: Effects on Child Development

**Published:** 1999

**Authors:** Joseph L. Jacobson, Sandra W. Jacobson

**Affiliations:** Joseph L. Jacobson, Ph.D., is a professor in the Department of Psychology, College of Science, and Sandra W. Jacobson, Ph.D., is a professor in the Department of Psychiatry and Behavioral Neurosciences, School of Medicine, Wayne State University, Detroit, Michigan

**Keywords:** moderate AOD use, gestation, prenatal alcohol exposure, growth retardation, behavioral problem, neurodevelopmental anomaly, intelligence and ability, fetal alcohol syndrome, amount of AOD use, child, mother, literature review

## Abstract

Children exposed to moderate levels of alcohol during pregnancy show growth deficits and intellectual and behavioral problems similar to, although less severe than, those found in children with fetal alcohol syndrome. Research has begun to examine the extent to which these problems affect the child’s ability to function on a day-to-day basis at school and with peers. Findings indicate that “ moderate” drinking has much more impact on child development when the mother consumes several drinks in a single day than when she drinks the same quantity in doses of one to two drinks per day over several days.

Moderate drinking^1^ during pregnancy is associated with developmental problems in childhood that resemble but are less severe than the growth deficiencies and intellectual and behavioral impairment found among children with fetal alcohol syndrome (FAS). Children with FAS grow more slowly than do other children both before and after birth, exhibit intellectual and social problems, and display a distinctive pattern of abnormal facial features (Jones and Smith 1973). Intellectual and behavioral impairment are the most disabling characteristics of FAS. About one-half of all FAS patients are mentally retarded (i.e., they have an IQ below 70^2^), and virtually all FAS patients exhibit serious attention and behavioral problems (Streissguth et al. 1991).

Several studies have found that children exposed to alcohol during pregnancy at lower levels than FAS children experience moderate intellectual and behavioral deficits that resemble those of FAS children but on a less severe level (Streissguth et al. 1993; Coles et al. 1997; Goldschmidt et al. 1996; J.L. Jacobson et al. 1996). Most of the mothers of children in these studies drank an average of 7 to 14 drinks per week (J.L. Jacobson and S.W. Jacobson 1994), a range generally considered as “moderate drinking.” Although the deficits associated with full-blown FAS are devastating, the more subtle developmental problems associated with lower levels of prenatal alcohol exposure are far more prevalent among children than FAS. In response, researchers at the Institute of Medicine (IOM) have suggested a new medical term—“alcohol-related neurodevelopmental disorder” (ARND)—characterized by the intellectual and behavioral deficits experienced in alcohol-exposed, non-FAS children (Stratton et al. 1996).

This article summarizes the effects of moderate prenatal alcohol exposure on children’s growth, intellectual competence, and behavior as well as discusses research findings regarding the impact of these effects on children’s general ability to function. The article also investigates the doses and patterns of moderate drinking during pregnancy that have been linked to developmental problems in offspring.

## Effects on Growth as well as Intellectual and Behavioral Function

Children whose mothers drink moderately during pregnancy exhibit growth deficits as well as intellectual and behavioral impairment.

### Growth

Although growth deficits are not a hallmark of ARND, consistent evidence indicates modest growth retardation in alcohol-exposed non-FAS infants before birth (e.g., Day et al. 1989; J.L. Jacobson et al. 1994*a*), and several studies have reported an association between prenatal alcohol exposure and slower-than-normal growth during the first 6 to 8 months after birth (J.L. Jacobson et al. 1994*b*). Moreover, deficits in height and head circumference have been documented in alcohol-exposed non-FAS children through age 6 (Day et al. 1994; also see Sampson et al. 1994). This slower growth pattern contrasts with the traditional finding that infants who weigh less at birth because of maternal smoking during pregnancy grow faster and tend to “catch up” during their first 5 to 6 months.

### Intellectual Function

Unlike children with FAS, who frequently have reduced IQ scores, non-FAS alcohol-exposed children do not necessarily demonstrate IQ deficits (e.g., Goldschmidt et al. 1996; Coles et al. 1997; also see Streissguth et al. 1993). For example, one study failed to find an overall IQ deficit among non-FAS alcohol-exposed children but found that they exhibited poorer arithmetic, reading, and spelling skills than did non-alcohol-exposed children (Goldschmidt et al. 1996). Researchers have documented arithmetic and attention deficits both in FAS children (Streissguth et al. 1991) and in at least three groups of children with ARND—(1) a group of predominantly white, middle-class children in Seattle who were prenatally exposed to moderate amounts of alcohol (Streissguth et al. 1993), (2) a group of economically disadvantaged African-American children in Detroit whose mothers drank moderately during pregnancy (S.W. Jacobson et al. 1993), and (3) a group of disadvantaged African-American children in Atlanta who were prenatally exposed to moderate-to-heavy amounts of alcohol (Coles et al. 1997).

To measure attention deficits, researchers commonly use tests for the four attention components identified by Mirsky and colleagues (1991) (see table 1). *Sustained attention* refers to the child’s ability to maintain focused concentration and alertness over time. *Focused attention* is a measure of the length of time the child maintains attention in the presence of distractions. *Executive function* involves the child’s ability to coordinate, plan, and execute appropriate responses and modify his or her behavior in response to feedback. *Working memory* is a measure of the child’s ability to mentally manipulate the information presented and to link this information with other information retrieved from memory.

Although research has documented low levels of sustained attention (Streissguth et al. 1993), focused attention (Streissguth et al. 1994), and executive function (Coles et al. 1997) in ARND children, these children’s most consistent deficits are in working memory. Streissguth and colleagues (1993) found that the two strongest negative effects observed in ARND children at age 7 were on arithmetic tests and the Digit Span test (which assesses the child’s ability to remember strings of digits); both are IQ subtests that depend most heavily on working memory. The two neuropsychological tests most strongly affected—the Children’s Memory Test (which assesses recall of details from stories read aloud to the child) and Seashore Rhythm (which assess the ability to discriminate between pairs of rhythmic patterns)—also measure working memory (Streissguth et al. 1993). Deficits on two working memory tests, the Stepping Stone Maze (which assesses the ability to find and recall an invisible path by moving a cursor through a matrix of squares) and Seashore Rhythm, also were among the strongest effects seen at age 14 (Streissguth et al. 1994). Similarly, S.W. Jacobson and colleagues (1998*b*) found that at age 7.5, the strongest effects were seen on arithmetic and Digit Span tests and timed tasks entailing mental manipulation of information, such as mental rotation (see table 1). Coles and colleagues (1997) also found that working memory impairments were among the strongest effects observed at 7.5 years.

Researchers have corroborated these effects on working memory in laboratory animal experiments, which have linked prenatal alcohol exposure to impaired performance on the Morris water maze (which assesses the animal’s ability to find and recall the location of a platform submerged in an opaque liquid) (Hannigan et al. 1993) and on the radial eight-arm maze (which assesses the animal’s ability to retrieve food pellets from the end of all eight arms of a maze without revisiting arms from which food has already been retrieved) at both moderate (Reyes et al. 1989) and heavy (Hall et al. 1994) levels of alcohol exposure.

### Behavioral Function

In addition to the intellectual and attention deficits found among non-FAS alcohol-exposed children, researchers also have documented behavior problems that resemble but are less severe than those found among FAS children. The socialization deficits associated with FAS include poor interpersonal skills and an inability to conform to social conventions (Streissguth 1997). Streissguth has described FAS patients as being “unaware of the consequences of [their] behavior, especially the social consequences,” showing “poor judgment in whom to trust,” and unable to “take a hint [i.e., needing strong clear commands]” (p. 127).

Relatively limited information is available regarding behavioral effects in alcohol-exposed non-FAS children. Using the Achenbach Child Behavior Checklist-Teacher’s Report Form (TRF), Brown and colleagues (1991) found poorer social competence and more aggressive and destructive behavior in children whose mothers drank throughout their pregnancies than in children whose mothers had stopped drinking in mid-pregnancy or abstained during pregnancy, independent of current maternal drinking patterns. In another study, prenatal alcohol exposure was associated with higher teacher ratings in three of the eight TRF problem areas—social, attention, and aggression—and greater inattention and impulsivity on the DuPaul-Barkley Attention Deficit Hyperactivity Disorder (ADHD) Scale, after controlling for potential confounding factors such as maternal smoking during pregnancy, quality of parenting, and current caregiver drinking (S.W. Jacobson et al. 1998*a*). Analyses showed that the social, aggression, and impulsivity problems were not merely by-products of the children’s attention deficits, indicating that alcohol directly affects diverse aspects of central nervous system function. A high proportion of children had problems in the borderline or clinical range. For example, 33 percent of the children prenatally exposed to moderate or heavy levels of alcohol exhibited aggressive behavior problems of this magnitude, compared with only 4 to 5 percent of the general population. One study found that at age 14, children with higher levels of prenatal alcohol exposure were more likely to have negative feelings about themselves; to be aggressive and delinquent; and to use alcohol, tobacco, and other drugs (Carmichael Olson et al. 1997).

## Effects on Children’s Day-to-Day Function

The effects of moderate prenatal alcohol exposure on children’s intellectual performance and behavior have been established. When examining the results of psychological tests, however, children with ARND often appear to have relatively subtle impairments (i.e., their average test scores are no more than a few points below normal). Although the *average* effect may be small, researchers have recently begun to examine whether the effects of moderate drinking are severe enough in certain children to affect their ability to manage on a day-to-day basis at school, home, and with peers.

To evaluate whether a specific deficit is severe enough to impair a child’s day-to-day function, researchers must establish criteria to indicate which test scores are low enough to be functionally significant (i.e., indicating a deficit severe enough to interfere with the ability to manage in school and other social contexts). For example, an IQ below 70 indicates mental retardation, but little consensus exists regarding the functional importance of a 5- or 10-point decrement when scores fall within the normal range. Moreover, for most psychological tests, such as those focusing on attention, no criteria for functional significance have been established, limiting the ability to evaluate the effect of ARND on children’s everyday function. In the absence of established criteria, Streissguth and colleagues (1993) used the bottom 7.5 percentile of scores to identify the children with the greatest deficiencies, and J.L. Jacobson and colleagues (1998) have used the bottom 10th percentile to indicate “poor performance.” These criteria are based on the premise that although the children’s performance at these levels may fall within the normal range, the performance levels are poor enough that they likely interfere with the children’s day-to-day functioning.

To determine whether the ARND children in one study had deficits that could be considered functionally significant, the researchers evaluated the children’s performance at approximately 12 months of age on four measures: (1) the Bayley Mental Index, which assesses simple fine motor and prehensile coordination (e.g., grasping a pencil and placing wood pieces in a puzzle) and imitation of a model; (2) the Bayley Psychomotor Index, which assesses walking and balance; (3) elicited play, which determines the most complex play with toys a child can imitate (e.g., placing a lid securely on a teapot or pretending to drink from a cup); and (4) cognitive processing speed, a measure of how quickly a child processes information, which is assessed by measuring the average length of the glances the child directs at an object or photograph (S.W. Jacobson et al. 1993). Children who scored in the bottom 10th percentile on a given outcome were considered to have a functionally significant deficit in that outcome.

The researchers then examined the association between the mothers’ alcohol consumption and the rates of functionally significant impairment in the children of both younger and older mothers (see figure). For the first three measures, mothers under age 30 did not appear to put their children at increased risk for functional impairment by drinking seven or more drinks per week (see J.L. Jacobson and colleagues [1998] for a discussion of the basis for the seven-drink-per-week threshold). For infants born to older women (i.e., age 30 and over), however, drinking above the threshold was associated with a three- to fivefold increase in functional impairment. For the fourth outcome, processing speed, drinking above the threshold doubled the risk of functional deficit in children of mothers in both age groups (i.e., the more heavily exposed children were statistically more likely to exhibit a functional deficit when the data in the two maternal age groups were pooled).

These findings are consistent with data from case studies of multiparous alcohol-abusing mothers of FAS children, which have shown that each successive child is almost always more severely impaired than the previous one. Similarly, animal experiments in which the doses of prenatal alcohol exposure were carefully controlled have documented markedly greater impairment in offspring born to older mothers.

**Figure:**
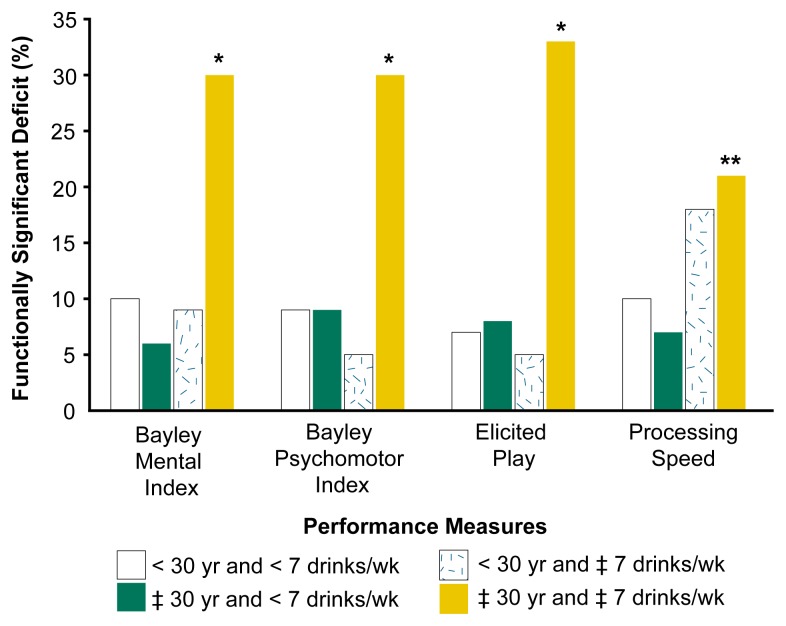
Rate of functionally significant deficit among offspring of older and younger mothers prenatally exposed to alcohol either above or below the threshold of seven drinks per week during pregnancy. None of the differences among the younger mothers were significant. Asterisks indicate significant differences between the offspring of the older mothers exposed above and below the threshold. *p < 0.01; **p < 0.10. yr = years; drinks/wk = drinks per week. SOURCE: J.L. Jacobson et al. 1996.

## Pattern of Maternal Drinking During Pregnancy

Most existing data on the effects seen in non-FAS alcohol-exposed children have been based on each of the mother’s alcohol intake averaged across her pregnancy. However, animal experiments indicate that this average is probably misleading, because ingesting a given dose of alcohol over a short time period (i.e., within a few hours) generates a greater peak blood alcohol concentration (BAC) and greater neuronal and behavioral impairment than does ingesting the same dose gradually over several days (Bonthius and West 1990).

The authors reanalyzed the infant data to examine the effects of dose (i.e., the average number of drinks per drinking occasion) and frequency (i.e., the average days per week of drinking during pregnancy) on developmental outcome (J.L. Jacobson et al. 1998). Children who scored in the bottom 10th percentile on one or more of the four outcomes in the figure were considered to be functionally impaired. As shown in table 2, 16 of the 20 functionally impaired infants (i.e., 80 percent) were born to women who drank on average at least five drinks per occasion during pregnancy (see table 2).

Ninety-one percent of the mothers in the study drank infrequently (i.e., no more than 2 days per week). Among the 11 infants in the sample whose mothers drank frequently (i.e., at least 4 days per week) during pregnancy, functional impairment was seen in 4 of the 5 infants whose mothers averaged at least 5 drinks per occasion but in none of the 6 infants whose mothers drank frequently at lower levels (ranging from 1.3 to 4.6 drinks per occasion). The one mother who drank daily was an alcoholic, and her infant was born with FAS. However, the infant of the frequently drinking mother who averaged only 1.3 drinks per occasion showed no evidence of neurodevelopmental impairment. The median drinking pattern of the mothers of the 20 children in table 2 with functional impairment was 7 drinks per occasion on 1 to 2 days per week. Although 7 to 14 drinks per week is often considered “moderate” drinking, this pattern of infrequent heavy doses may be characterized more accurately as heavy weekend drinking.

## Conclusions

Several studies have found that moderate prenatal alcohol exposure has statistically significant effects on children’s cognitive and behavioral development. Using the IOM-proposed terminology, many of these children would be diagnosed as having ARND. ARND differs from FAS, however, in that FAS is characterized by reduced IQ scores and more severe socialization problems. Nevertheless, evaluations of the specific domains in which deficits occur reveal important parallels between FAS and ARND. In the cognitive domain, arithmetic, attention, and working memory are most severely and consistently affected in both disorders. In the behavioral domain, both disorders are marked by increased impulsivity, aggression, and social problems. Researchers are only beginning to address the importance of these deficits for the day-to-day functioning of the ARND child. The aforementioned data suggest that although some non-FAS alcohol-exposed children are only minimally affected by prenatal alcohol exposure, other more susceptible children are impaired to a degree likely to interfere with their ability to function normally. Detailed information about the functional significance of each of the deficits found among ARND children is needed to fully understand the implications of prenatal alcohol exposure for child development.

More attention also should be devoted to determining the specific drinking levels and patterns associated with functionally significant developmental impairment. Research has documented functionally significant deficits in infants whose mothers drank, on average, five or more drinks per occasion once or twice per week. Although considered excessive for a pregnant woman, this level of drinking falls short of the rate usually associated with having a serious drinking problem. Given the marked individual differences in alcohol metabolism and fetal vulnerability, five drinks per occasion may be too high a threshold for many women. Functional deficits may occur in some children who are repeatedly exposed prenatally to only three or four drinks per occasion, especially if the alcohol is consumed on an empty stomach. In evaluating the risk associated with exposure to environmental and food contaminants, a safety margin is usually incorporated to allow for individual differences in sensitivity. Where human data are available, a safety factor of 10 is used for this purpose (Sette and Levine 1986). Using this approach, researchers might divide the threshold value of seven drinks per week that is often found for the neurobehavioral effects of alcohol (J.L. Jacobson and S.W. Jacobson 1994) by 10 and conclude that 0.7 drinks per week (one drink every 10 days) is likely considered to be “safe” drinking. Obstetrical care providers can reassure patients who have consumed a few alcoholic beverages once or twice early in their pregnancies that they need not be overly concerned.

These data demonstrate a statistically significant association between moderate drinking during pregnancy and children’s adverse neurobehavioral outcomes. The data also demonstrate that these effects may be severe enough in some children to affect their day-to-day functioning. Although children exposed to moderate levels of alcohol during pregnancy are not mentally retarded, they show attention deficits and behavioral problems that are similar to, although less severe than, those found in FAS children. These data also demonstrate that as with most neurotoxicants, the human organism is markedly more vulnerable to alcohol exposure during the prenatal period than at any other point in the lifespan. Because of this heightened vulnerability and the apparently long-term, permanent nature of alcohol-related deficits, the best advice continues to be abstinence or, at most, minimal consumption of alcohol during pregnancy.

## Figures and Tables

**Table 1:** Four Dimensions of Attention

Dimension of Attention	Definition	Relevant Task	Description of Task
Sustained attention	Ability to maintain focus and alertness	Continuous Performance Test	A series of letters is displayed on a computer screen, and the child presses a button whenever a pre-designated target stimulus appears.
Focused attention	Ability to maintain attention in the presence of distractions	Digit Cancellation (interference condition)	The child crosses out all “3s” and “7s” on a page of random digits while hearing strings of numbers read aloud through headphones.
Executive function	Ability to coordinate, plan, and execute appropriate responses	Tower of London	The child is presented with a small board with three pegs varying in height and three beads varying in color. The child tries to reorder the beads on the pegs in a limited number of moves to conform to the arrangement shown in a drawing.
Working memory	Sequential mental manipulation of information linking input from the environment with information retrieved from memory	Mental Rotation	The child determines within a limited period of time whether a letter displayed at one of several angles (e.g., 30°, 60°) is forward or backward (i.e., a mirror image).

**Table 2:** Relation of Alcohol Dose per Occasion During Pregnancy to Incidence of Functionally Significant Deficit in Offspring During Infancy*

Functional Deficit	Drinks per Occasion	

	< 5	≥ 5
Yes	4 (21%)	16 (57%)
No	15 (79%)	12 (43%)
	
Total children	19 (100%)	28 (100%)

* *N* = 47.

NOTES: Only mothers who averaged at least seven drinks per week during pregnancy were included in this analysis. The relation shown in the table is statistically significant at *p* < 0.025.

SOURCE: J.L. Jacobson et al. 1998.

## Footnotes

1 Definitions of moderate drinking vary. For the purposes of this article, 7 to 14 drinks per week is considered moderate drinking. The Federal government has adopted more conservative guidelines, defining moderate drinking as no more than one drink per day for women and no more than two drinks per day for men (U.S. Department of Agriculture and the U.S. Department of Health and Human Services 1995).

2 Most IQ tests have a mean of 100 (i.e., 100 is considered normal) and a standard deviation of 15 points. An IQ of less than 70 is usually considered to indicate mental retardation, and IQ scores of 70 to 79 are considered to indicate borderline mental retardation.
